# Life expectancy after bariatric surgery or usual care in patients with or without baseline type 2 diabetes in Swedish Obese Subjects

**DOI:** 10.1038/s41366-023-01332-2

**Published:** 2023-07-12

**Authors:** Lena M. S. Carlsson, Björn Carlsson, Peter Jacobson, Cecilia Karlsson, Johanna C. Andersson-Assarsson, Felipe M. Kristensson, Sofie Ahlin, Per-Arne Svensson, Magdalena Taube, Ingmar Näslund, Kristjan Karason, Markku Peltonen, Kajsa Sjöholm

**Affiliations:** 1 Institute of Medicine, Sahlgrenska Academy, University of Gothenburg, Gothenburg, Sweden; 2 Research and Early Development, Cardiovascular, Renal and Metabolism (CVRM), BioPharmaceuticals R&D, AstraZeneca, Gothenburg, Sweden; 3 Late-Stage Development, Cardiovascular, Renal and Metabolism (CVRM), BioPharmaceuticals R&D, AstraZeneca, Gothenburg, Sweden; 4 Region Västra Götaland, Department of Oncology, Sahlgrenska University Hospital, Gothenburg, Sweden; 5 Region Västra Götaland, Department of Clinical Physiology, NU Hospital Group, Trollhättan, Sweden; 6 Institute of Health and Care Sciences, Sahlgrenska Academy, University of Gothenburg, Gothenburg, Sweden; 7 Department of Surgery, Faculty of Medicine and Health, Örebro University, Örebro, Sweden; 8 Finnish Institute for Health and Welfare, Helsinki, Finland

**Keywords:** Diabetes, Type 2 diabetes, Obesity, Cardiovascular diseases

## Abstract

**Objectives:**

To determine life expectancy and causes of death after bariatric surgery in relation to baseline type 2 diabetes (T2D) in the prospective, Swedish Obese Subjects study.

**Methods:**

The study included 2010 patients with obesity who underwent bariatric surgery and 2037 matched controls, eligible for surgery. The surgery group underwent gastric bypass (*n* = 265), banding (*n* = 376), or vertical banded gastroplasty (*n* = 1369). The control group (*n* = 2037) received usual obesity care. Causes of death were obtained from the Swedish Cause of Death Register, case sheets and autopsy reports, in patients with baseline T2D (*n* = 392 surgery patients/*n* = 305 controls) or non-T2D (*n* = 1609 surgery patients/*n* = 1726 controls) during a median follow-up 26 years.

**Results:**

In T2D and non-T2D subgroups, bariatric surgery was associated with increased life expectancy (2.1, 95% confidence interval (95% CI) 0.2–4.0; and 1.6, 0.5–2.7 years, respectively) and reduced overall mortality (adjusted hazard ratio (adjHR) = 0.77, 95% CI: 0.61–0.97; and 0.82, 0.72–0.94, respectively), and the treatment benefit was similar (interaction *p* = 0.615). Bariatric surgery was associated with reduced cardiovascular mortality in both subgroups (adjHR = 0.65, 95% CI: 0.46–0.91; and 0.70, 0.55–0.88, respectively (interaction *p* = 0.516)).

**Conclusions:**

Bariatric surgery is associated with similar reduction of overall and cardiovascular mortality and increased life expectancy regardless of baseline diabetes status.

## Introduction

Obesity, a chronic disease that affects a large part of the population worldwide, is associated with reduced life expectancy. Patients with obesity have increased risk of severe diseases, including type 2 diabetes (T2D) and cardiovascular disease, and those with both obesity and T2D have the highest risk of cardiovascular events [1]. Achieving and maintaining a healthy body weight is needed to prevent obesity-related diseases and mortality. Bariatric surgery is an established treatment of obesity and results in large, sustained weight loss, as well as improvement of cardiovascular risk factors, including remission or prevention of T2D depending on diabetes status at the time of surgery [2–4].

Several studies have shown that bariatric surgery is associated with reduced overall mortality in patients with obesity [5–8]. Moreover, some studies suggest that the treatment benefit may differ depending on diabetes status at the time of surgery [9] but others do not [7, 10]. So far, few studies have examined to which extent the reduced relative risk of mortality after bariatric surgery increases life expectancy. In the prospective Swedish Obese Subjects (SOS) study, which includes both patients with and without pre-existing T2D, we recently reported that life expectancy was approximately 3 years longer after bariatric surgery compared to usual obesity care with a median follow-up exceeding 20 years and, that there was no interaction with classical risk factors, including glucose tolerance [11]. Also, a recent retrospective report from the Utah population database with a median follow-up of 11 years suggests a 1.3-year longer restricted mean survival time after bariatric surgery [8]. In contrast, a meta-analysis published in 2021 suggests that bariatric surgery on average increases life expectancy by 9.3 years in patients with diabetes but only by 5.1 years in patients without diabetes [12]. Notably, the meta-analysis is almost exclusively based on retrospective data with relatively short follow-up (median 6 years) and control patients captured from registers with limited information on health status.

Many patients with obesity that consider bariatric surgery are motivated by the possibility that this treatment may reduce the risk of premature death [13]. The large discrepancies between the results from our prospective SOS study and the meta-analysis could affect their decisions. We therefore extend our previous analysis of life expectancy after bariatric surgery [11] with even longer follow-up, and include a more detailed analysis of cause-specific mortality in subgroups with and without baseline T2D. Importantly, comparisons are made with contemporaneously matched controls known to be eligible for surgery.

## Research design and methods

**Fig. 1:**
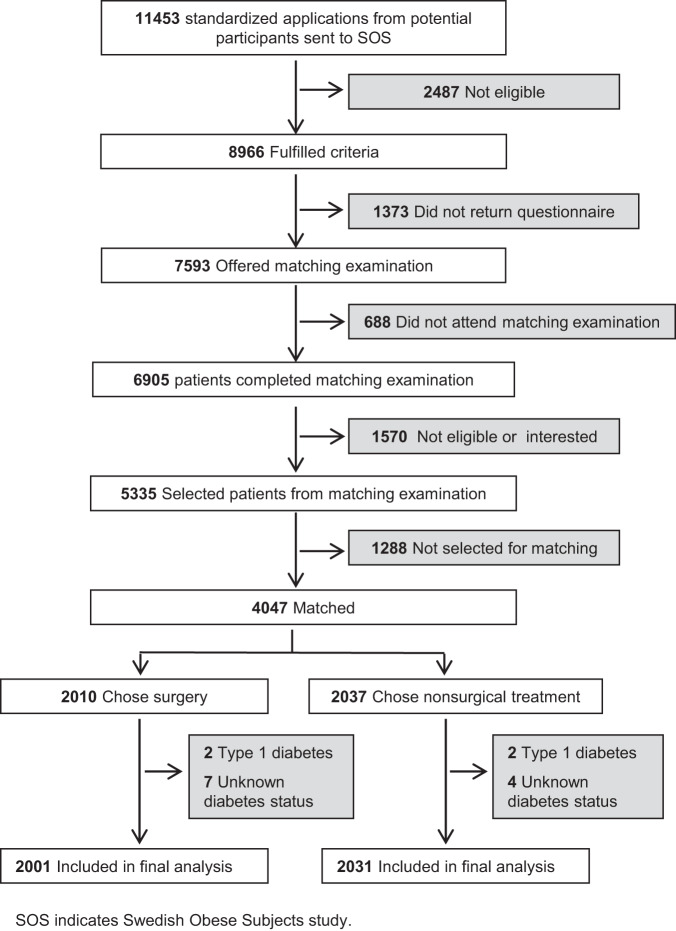
Trial flow diagram. The trial flow diagram shows the selection of participants for the SOS study and reasons for exclusion in the current analysis.

The SOS study, conducted at 25 surgical departments and 480 primary healthcare centers in Sweden, has previously been described [5, 11]. The trial flow diagram is presented in Fig. 1 and details on study design are given in the Appendix. Patients were included between September 1, 1987, and January 31, 2001, and the study consists of a surgery group (*n* = 2010) with patients who chose surgical treatment and a contemporaneously matched control group (*n* = 2037) with identical inclusion and exclusion criteria. The inclusion criteria were age 37–60 years and body mass index (BMI) of ≥34 kg/m^2^ for men and ≥38 kg/m^2^ for women and all participants were eligible for surgery. The exclusion criteria were established to exclude patients with unacceptable surgical risks. Surgery patients underwent gastric bypass, banding or vertical banded gastroplasty. The type of surgery was determined by surgeons at the participating surgical departments. Patients in the control group received conventional obesity and diabetes care at their primary healthcare center. The study began on the day of surgery for patients in the surgery group as well as for their matched controls. Examinations were performed at baseline and after 0.5 and 1, 2, 3, 4, 6, 8, 10, 15 and 20 years.

Fasting blood samples were taken at baseline, and after 2, 10, 15 and 20 years. From 1987 to 2009, glucose concentrations were measured in venous whole blood at the Central Laboratory, Sahlgrenska University Hospital, accredited according to ISO/IEC 189. After August 1, 2009, venous plasma glucose has been measured and converted to blood glucose according to the instructions from the Central Laboratory (blood glucose = plasma glucose / 1.12). The SOS study was started before repeated measurements were routinely used for the diagnosis of T2D, and single determinations were therefore used for diagnosis. Self-reported diabetes and hypertension medication was obtained from SOS questionnaires.

Diabetes was defined by the use of diabetes medication, a fasting blood glucose level of ≥6.1 mmol/L (corresponding to fasting plasma glucose of ≥7.0 mmol/L) or HbA1c ≥48 mmol/mol [14]. In patients with diabetes onset before the age of 35, we excluded those that were positive for glutamate decarboxylase antibodies or islet cell antibodies or with C-peptide values below the detection limit to rule out type 1 diabetes and latent autoimmune diabetes in adults. Patients where diabetes status at baseline could not be determined due to missing data were also excluded.

The SOS database was crosschecked against the Swedish Population Register to obtain information on all deaths until December 31, 2020. Patients who emigrated (*n* = 54), withdrew consent (*n* = 3), or were alive at the end of follow-up (*n* = 2698) were censored on the corresponding date. The official (underlying) cause of death was obtained from the Swedish Cause of Death Register. In addition, relevant case sheets and autopsy reports were assessed independently by two authors who were unaware of the patient’s study-group assignment to determine the direct cause of death. If the study-determined cause of death and the official cause of death differed, the study-determined direct cause of death was used [11].

Seven Swedish regional ethics review boards approved the study protocol, and written or oral informed consent was obtained from all patients. This study is registered at ClinicalTrials.gov (NCT01479452).

### Statistical methods

Data are presented as mean values with standard deviations or as percentages. Baseline comparisons between the groups were performed with an analysis of covariance for continuous variables and with Fisher’s exact test or a logistic regression model for dichotomous variables. Patients were included in the analysis according to intention to treat. A sensitivity analysis was done based on as treated (per-protocol). We used the Gompertz proportional hazards regression model to compare mortality and life expectancy in the surgery and control groups [11]. The results are presented as hazard ratios for death and differences in estimated median life expectancy between the groups with corresponding confidence intervals. Analyses of cause-specific mortality were conducted with the competing-risks regression models suggested by Fine and Gray [15], in which deaths for other reasons were treated as competing events. Adjusted analyses included adjustment for preselected predictors (age, sex, BMI and smoking at baseline, and year of inclusion in the study). All statistical tests were two-sided, and *p* values of less than 0.05 were considered to indicate statistical significance. Stata software, version 15.1 (StataCorp), was used for all analyses.

## Results

Overall, 4032 of the 4047 individuals in the SOS study were included in the current analysis. Four patients with type 1 diabetes and 11 patients with missing information on diabetes status at baseline were excluded (Fig. 1). Mortality was separately analyzed in 392 surgery patients and 305 controls with T2D, and 1609 surgery patients and 1726 controls without T2D (non-T2D), respectively. The median follow-up time was 26.1 years (interquartile range 22.7–28.6 years) and 25.9 years (interquartile range 22.7–28.3 years) for the T2D and non-T2D subgroups, respectively.

Table 1 shows baseline characteristics in T2D and non-T2D SOS participants, stratified by intervention. Regardless of baseline diabetes status, controls were slightly older, while BMI was higher and most risk factors less favorable in surgery patients. BMI during 20 years of follow-up is shown in Supplementary Fig. 1. After bariatric surgery, average BMI was lowest at the 1-year follow-up in both the T2D and non-T2D subgroups (32.6 and 31.6 kg/m^2^, respectively), after which partial weight regain occurred. In the controls, BMI changes were smaller and with a decrease in the T2D and an increase in the non-T2D subgroup (Supplementary Fig. 1).

**Table 1:** Characteristics of the surgery and control groups of the SOS study stratified by baseline diabetes status.

	T2D			Non-T2D		
Characteristic	Surgery^a^ (*n* = 392)	Control (*n* = 305)	*p* value^b^	Surgery^c^ (*n* = 1609)	Control (*n* = 1726)	*p* value^b^
Male sex, *n* (%)	157 (40.1)	125 (41.0)	0.816^d^	431 (26.8)	464 (26.9)	0.969^d^
Age, years	48.6 ± 6.0	50.5 ± 6.3	<0.001	46.8 ± 5.9	48.4 ± 6.2	<0.001
Body weight, kg	123.6 ± 19.1	116.8 ± 16.8	<0.001	120.3 ± 15.9	114.4 ± 16.5	<0.001
BMI, kg/m^2^	42.4 ± 4.9	40.1 ± 4.7	<0.001	42.4 ± 4.4	40.1 ± 4.7	<0.001
Waist circumference, cm	129.0 ± 11.8	123.3 ± 9.9	<0.001	125.0 ± 10.6	119.7 ± 11.4	<0.001
Waist-to-hip ratio	1.021 ± 0.079	1.012 ± 0.065	0.112	0.986 ± 0.076	0.972 ± 0.074	<0.001
Cholesterol, mmol/L	5.95 ± 1.26	5.68 ± 1.14	0.004	5.84 ± 1.09	5.60 ± 1.04	<0.001
HDL cholesterol, mmol/L	1.25 ± 0.30	1.23 ± 0.28	0.376	1.37 ± 0.32	1.37 ± 0.33	0.731
LDL cholesterol, mmol/L	3.41 ± 1.03	3.25 ± 1.03	0.054	3.53 ± 0.94	3.38 ± 0.90	<0.001
Non-HDL cholesterol, mmol/L	4.58 ± 1.15	4.41 ± 1.17	0.054	4.45 ± 1.08	4.22 ± 1.03	<0.001
Blood glucose, mmol/L	8.24 ± 2.72	8.16 ± 2.72	0.700	4.43 ± 0.58	4.36 ± 0.59	0.001
Serum insulin, pmol/l	170.2 ± 113.1	151.3 ± 102.3	0.021	119.4 ± 69.6	100.6 ± 57.3	<0.001
HbA1c, mmol/mol	61.5 ± 16.6	60.2 ± 15.5	0.324	37.6 ± 3.9	37.0 ± 3.9	<0.001
HbA1c, %	7.8 ± 1.5	7.7 ± 1.4	0.324	5.59 ± 0.36	5.54 ± 0.36	<0.001
HOMA-IR	11.6 ± 8.7	10.2 ± 8.1	0.023	4.5 ± 2.9	3.7 ± 2.4	<0.001
Prediabetes, *n* (%)^e^	–	–	–	570 (35.4)	490 (28.4)	<0.001^d^
Systolic blood pressure, mmHg	150.0 ± 18.9	143.3 ± 18.5	<0.001	143.8 ± 18.5	137.0 ± 17.7	<0.001
Diastolic blood pressure, mmHg	91.6 ± 11.2	87.4 ± 11.1	<0.001	89.5 ± 11.1	84.8 ± 10.5	<0.001
Hypertension, *n* (%)^f^	347 (88.5)	251 (82.3)	0.022^d^	1220 (76.0)	1046 (60.7)	<0.001^d^
University education, *n* (%)	46 (11.7)	54 (17.7)	0.029^d^	211 (13.1)	376 (21.8)	<0.001^d^
Smoking daily, *n* (%)	100 (25.6)	66 (22.0)	0.283^d^	416 (25.9)	354 (20.6)	<0.001^d^
Alcohol use, g/day	5.03 ± 7.75	5.59 ± 8.69	0.420	5.18 ± 7.29	5.47 ± 8.47	0.326
Previous cancer, *n* (%)^g^	7 (1.8)	6 (2.0)	1.000^d^	18 (1.1)	15 (0.9)	0.489^d^
Previous cardiovascular disease, *n* (%)^g^	21 (5.4)	15 (4.9)	0.864^d^	25 (1.6)	33 (1.9)	0.508^d^
SCORE, 10-year risk^h^	2.37 ± 2.91	2.52 ± 3.10	0.530	1.45 ± 2.08	1.43 ± 2.01	0.839
Year of inclusion	1994.0 ± 3.5	1994.2 ± 3.6	0.512	1994.1 ± 3.3	1994.4 ± 3.4	0.023
2-year remission, *n* (%)	247 (71.6)	42 (16.9)	<0.001^d^	–	–	–

Data are mean (SD) or *n* (%).

*T2D* type 2 diabetes.

^a^Surgical treatment: *n* = 257 vertical banded gastroplasty, *n* = 68 banding, *n* = 67 gastric bypass.

^b^Difference between surgery and control groups.

^c^Surgical treatment: *n* = 1110 vertical banded gastroplasty, *n* = 304 banding, *n* = 195 gastric bypass.

^d^Fischer’s exact test for categorial variables.

^e^Defined as impaired fasting glucose (plasma glucose 5.6–6.9 mmol/l) OR elevated HbA1c (39–47 mmol/mol (5.7–6.4%)).

^f^Defined as diastolic blood pressure >90 mmHg, systolic blood pressure >140 mmHg, or self-reported antihypertensive medication.

^g^Self-reported data from SOS questionnaire.

^h^European SCORE (Systematic Coronary Risk Evaluation) model (Eur Heart J. 2003;24:987–1003).

### Overall mortality and life expectancy

**Fig. 2:**
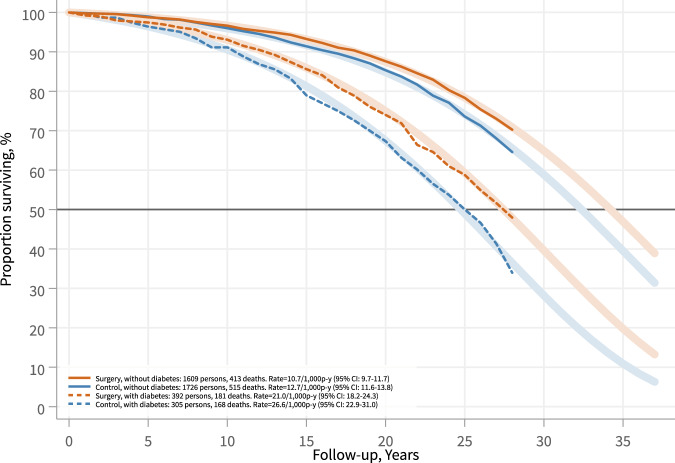
Survival in the surgery and control groups stratified by baseline type 2 diabetes status. Shown are subgroups with (dashed lines) and without (solid lines) baseline diabetes. The graph shows the Kaplan–Meier estimate of survival (opaque lines) and the survival estimated from an unadjusted Gompertz regression model extrapolated up to 37.5 years (fainter lines). Intention-to-treat analysis. p-y, person-years.

Figure 2 shows cumulative survival rates and predicted survival based on a model of the time to death. The adjusted difference in median life expectancy in the T2D compared to the non-T2D subgroup was –5.3 (95% CI: –6.9 to –3.8) years in usual care controls and –4.8 (95% CI: –6.3 to –3.3) years in surgery patients. Bariatric surgery was associated with increased life expectancy and lower overall mortality in both the T2D (adjusted hazard ratio [adjHR], 0.77, 95% CI: 0.61–0.97; *p* = 0.026) and the non-T2D subgroup (0.82, 95% CI: 0.72–0.94; *p* = 0.004), and the surgical treatment benefit was not related to diabetes status at baseline (*p* for interaction 0.615) (Table 2).

**Table 2:** Mortality rates, hazard ratios and differences in median life expectancy from Gompertz proportional hazard regression model.

	T2D		Non-T2D	
	Control	Surgery	Control	Surgery
*n*	305	392	1726	1609
Person-years	6308	8600	40,639	38,776
Events	168	181	515	413
Mortality rate per 1000 person-years (95% CI)	26.6 (22.9–31.0)	21.0 (18.2–24.3)	12.7 (11.6–13.8)	10.7 (9.7–11.7)
HR (95% CI) (adjusted^a^)	0.77 (0.61–0.97)		0.82 (0.72–0.94)	
*p* value	0.026		0.004	
Interaction, *p* value (adjusted^a^)	0.615			
Difference in median survival time (95% CI), years (unadjusted)	2.7 (0.9–4.6)		1.8 (0.7–3.0)	
Difference in median survival time (95% CI), years (adjusted^a^)	2.1 (0.2–4.0)		1.6 (0.5–2.7)	

*T2D* type 2 diabetes.

^a^Adjusted for sex, age, BMI and smoking at baseline, and year of inclusion.

### Cause-specific mortality

**Fig. 3:**
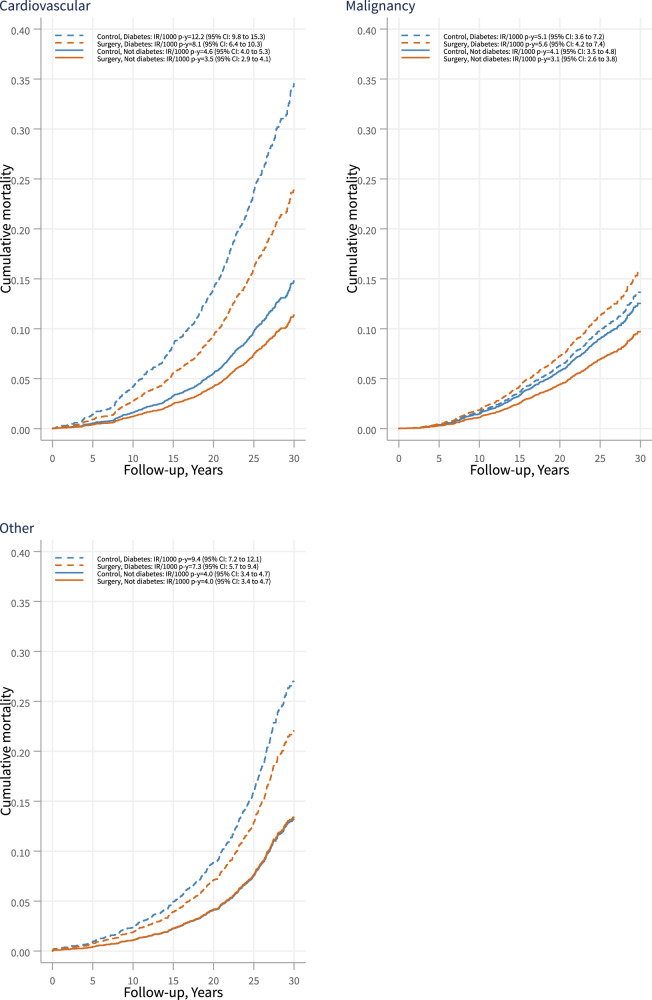
Cause-specific mortality in the surgery and control groups stratified by baseline diabetes status. Shown are subgroups with (dashed lines) and without (solid lines) baseline type 2 diabetes. IR incidence rate, p-y person-years.

Cardiovascular diseases and malignancies were the most common causes of death in both the T2D and the non-T2D subgroup (Supplementary Table 1). Figure 3 shows cumulative mortality due to cardiovascular disease, malignancy or other causes. Regardless of treatment, fatal cardiovascular events were more common in patients with baseline diabetes compared with those without diabetes. In both the T2D and the non-T2D subgroups, bariatric surgery was associated with reduced cardiovascular mortality (adjHR = 0.65, 0.46–0.91; *p* = 0.012 and adjHR = 0.70, 0.55–0.88; *p* = 0.003, respectively), while there were no significant associations with deaths caused by malignancies or other causes (Supplementary Table 1). When deaths caused by specific cardiovascular diseases were analyzed, bariatric surgery was associated with reduced incidence of fatal cardiac disease (myocardial infarction, heart failure, sudden death and other cardiac disease) in both subgroups while no association with stroke was observed in either subgroup (Supplementary Table 1). There was a difference in surgical treatment effect for fatal myocardial infarction between diabetes subgroups (adjusted *p* for interaction 0.013), and an association between surgery and reduced incidence was only observed in patients with T2D (Supplementary Table 1). We found no differences in treatment effects (i.e., interactions were non-significant) between diabetes subgroups with respect to cancer-related death or overall death from other causes (Supplementary Table 1). Similar results were obtained in sensitivity analyses based on as treated T2D and non-T2D subgroups (Supplementary Fig. 2).

## Conclusions

In this study we examined the association between bariatric surgery and life expectancy in patients with and without diabetes, using long-term follow-up data from the prospective controlled SOS study. We found that bariatric surgery was associated with a similar increase in life expectancy in patients with and without baseline T2D. Our results differ substantially from a recent meta-analysis, both with respect to overall survival benefit and differences in survival benefit between patients with and without T2D [12]. The authors of the meta-analysis used the modeling technique previously used in the SOS study [11] but the diverging results could be due to important differences with respect to study design, the quality of underlying data and length of follow-up.

Life expectancy is reduced in patients with obesity and further reduced in those with concomitant T2D, as observed here and elsewhere [16, 17]. However, only the SOS study, the recent retrospective study from the Utah population database [8] and the meta-analysis which is based on 16 retrospective studies and one prospective (i.e., the SOS study) [12] have previously examined the effect of bariatric surgery on life expectancy. In the meta-analysis, the overall estimated increase in median life expectancy after bariatric surgery was 6.1 years [12], which is considerably longer than the increase observed in our previous report from the SOS study (3.0 years) [11]. There could be several different explanations for this discrepancy but the most important is probably that the meta-analysis relies on retrospective data whereas our study is prospective. In the SOS study, surgically and non-surgically treated groups are similar due to identical inclusion and exclusion criteria and extensive subsequent matching for factors hypothesized to influence survival, while much less is known about possible differences between treatment groups in the retrospective studies included in the meta-analysis. For example diabetes duration, smoking status and other factors affecting mortality may be unknown in retrospective studies. Importantly, in our study, both surgery patients and usual care controls were eligible for surgery and such information is rarely available for retrospective studies. Furthermore, controls with obesity identified from patient registers may have other diseases as the primary cause for hospitalization. If controls are less healthy than surgically treated subjects, the overall effect of surgery would be overestimated. On the other hand, controls with obesity included in an intervention study such as the SOS study may be prone to a healthier life style since their body weights are monitored at regular intervals, and this could explain the more modest benefit of bariatric surgery in our study.

Another important factor that differs between the studies is the length of follow-up. Survival estimates in the meta-analysis were based on an observed median follow-up of 5.8 years, and a considerable extrapolation over time was therefore necessary to get an estimate of median life expectancy; i.e., survival until 50% of the participants have died. Thus, extensive length of follow-up or extensive extrapolation is required. In this report, the estimated follow-up time until half of the participants had died was approximately 27 years in patients with diabetes and 34 years in patients without diabetes, and the follow-up needed in the meta-analysis was in the same range [12]. Notably, with the current length of follow-up in the SOS study, more than half of the patients with baseline diabetes have died, suggesting that our estimates of median life expectancy may be more reliable.

In the current report we were unable to detect a difference in life-years gained in those with and without T2D while the meta-analysis suggests that the treatment benefit of bariatric surgery is substantially larger in patients with T2D (9.3 years), compared to patients without diabetes (5.1 years). Nevertheless, a more recent, large retrospective study that ensured that controls satisfied the eligibility criteria used for surgery patients, found that the 5-year mortality benefit was similar in subgroups defined by baseline diabetes status [7], which is in line with our findings. For patients with baseline diabetes, treatment benefit of bariatric surgery could partly depend on pre-existing irreversible organ damage which is more common in patients with long diabetes duration [3, 18]. In the SOS study, surgery and control groups have similar diabetes duration [3], while this information is not taken into account in the meta-analysis. If patients in the control group have more severe diabetes compared to patients in the surgery group, this could result in an overestimation of the effects of bariatric surgery on estimated survival benefit. Furthermore, treatment benefit of bariatric surgery in patients with diabetes could also partly depend on whether short-term or durable diabetes remission is achieved, which is also affected by the severity and duration of diabetes [3, 18].

We and others have previously reported associations between bariatric surgery and reduced overall, CVD-related, and cancer-related mortality in patients with obesity [6, 7, 11, 12, 19, 20]. In this report, we extend these findings by reporting cause-specific mortality by baseline diabetes status. We found that the relative risk of cardiovascular death was reduced to a similar degree after bariatric surgery in patients with and without baseline diabetes. However, patients with baseline diabetes were less likely to die from myocardial infarction after surgery. We found no significant associations between bariatric surgery and cancer death in patients with or without baseline diabetes. However, it should be noted that number of cause-specific deaths, including cancer, was relatively low in patients with diabetes, and therefore the observed results should be interpreted with care.

A major strength of the SOS study is the virtually complete, very long-term follow-up of surgically treated patients and matched controls treated with usual care. Another important strength is the extensive information about the usual care controls which ensures that they are healthy enough to be eligible for surgery. This study also has several limitations. One limitation is that the study was not randomized due to the high risk of bariatric surgery in the 1980s and in spite of careful matching there are still some remaining differences between the treatment groups. However, given the fact that several studies indicate that bariatric surgery is associated with reduced mortality it is, for ethical reasons, unlikely that large randomized long-term studies designed to analyze mortality will be initiated. Another limitation is that most patients were treated with surgical procedures that are not used today.

In conclusion, our prospective study indicates that bariatric surgery is associated with a similar increase in life expectancy in patients with and without T2D and that the survival benefit is much more modest compared to previous estimates based on retrospective data. Although our results and results from the meta-analysis indicates a survival benefit for patients treated with bariatric surgery, the magnitude of the effect differs substantially and could influence the decision process for patients considering bariatric surgery.

### Supplementary information

Supplementary information

## Data Availability

Restrictions apply to the general availability of the data because of patient agreements and the nature of the data.
