# Supplementary material for: Life expectancy after bariatric surgery or usual care in patients with or without baseline type 2 diabetes in Swedish Obese Subjects
Source: Int J Obes (Lond). 2023 Jul 12;47(10):931–8. doi: 10.1038/s41366-023-01332-2 (PMC10511310; doi:10.1038/s41366-023-01332-2)
Supplement: Supplementary file 1 — Supplementary information [file 41366_2023_1332_MOESM1_ESM.docx]

# Supplementary appendix

##### Study design

The SOS study, conducted at 25 surgical departments and 480 primary healthcare centers in Sweden, consists of a surgery group (n=2010) with participants who chose surgical treatment and a contemporaneously matched control group (n=2037). The surgery and controls groups were matched with respect to 18 matching variables that may affect prognosis. The matching variables were sex, age, weight, height, waist and hip circumferences, systolic blood pressure, serum cholesterol and triglyceride levels, smoking status, diabetes, menopausal status, four psychosocial variables with documented associations with the risk of death, and two personality traits related to treatment preferences. The surgery and control groups had identical inclusion and exclusion criteria. The inclusion criteria were age 37 to 60 years and body mass index (BMI) of 34 kg/m^2^ or more for men and 38 kg/m^2^ or more for women. The exclusion criteria were earlier surgery for gastric or duodenal ulcer, earlier bariatric surgery, gastric ulcer during the past 6 months, ongoing malignancy, active malignancy during the past 5 years, myocardial infarction during the past 6 months, bulimic eating pattern, drug or alcohol abuse, psychiatric or cooperative problems contraindicating bariatric surgery, other contraindicating conditions (eg chronic glucocorticoid or anti-inflammatory treatment).

##### sTables

**sTable 1.** Causes of death in the T2D and non-T2D groups, stratified by intervention.

|  | **Type 2 diabetes** | | | **No type 2 diabetes** | | |  |
| --- | --- | --- | --- | --- | --- | --- | --- |
|  | **Surgery N=392** | **Control N=305** | **Adj. sHR (95% CI)**^1^ | **Surgery N=1609** | **Control N=1726** | **Adj. sHR (95% CI)** ^1^ | **Adj. p for interaction**^1^ |
| **CARDIOVASCULAR** | **70** | **77** | **0.65 (0.46-0.91)** | **135** | **186** | **0.70 (0.55-0.88)** | **0.516** |
| **CARDIAC** | 59 | 67 | 0.64 (0.44-0.93) | 115 | 155 | 0.71 (0.55-0.92) | 0.428 |
| MI | 12 | 30 | 0.37 (0.18-0.75) | 29 | 33 | 0.88 (0.51-1.51) | 0.013 |
| Heart failure | 12 | 10 | 0.80 (0.34-1.85) | 15 | 39 | 0.40 (0.22-0.74) | 0.122 |
| Sudden death | 34 | 27 | 0.88 (0.51-1.53) | 68 | 77 | 0.83 (0.59-1.17) | 0.990 |
| Other cardiac | 1 | 0 |  | 3 | 6 | 0.65 (0.15-2.70) |  |
| **STROKE** | 9 | 10 | 0.69 (0.29-1.68) | 17 | 24 | 0.80 (0.42-1.52) | 0.936 |
| **OTHER CARDIOVASCULAR**^2^ | 2 | 0 |  | 3 | 7 | 0.43 (0.10-1.89) |  |
| **MALIGNANCY** | **48** | **32** | **1.31 (0.82-2.08)** | **122** | **167** | **0.80 (0.63-1.02)** | **0.092** |
| **OTHER** | **63** | **59** | **0.98 (0.66-1.46)** | **156** | **162** | **1.06 (0.85-1.33)** | **0.383** |
| Benign tumor | 0 | 0 |  | 0 | 1 |  |  |
| Complications after bariatric surgery | 3 | 0 |  | 4 | 0 |  |  |
| Complications after other surgery | 3 | 1 |  | 8 | 6 | 1.40 (0.50-3.91) | 0.685 |
| Infection | 18 | 21 | 0.66 (0.34-1.29) | 38 | 50 | 0.88 (0.56-1.38) | 0.589 |
| Neurological disease | 5 | 4 | 1.27 (0.27-6.09) | 14 | 14 | 1.20 (0.55-2.60) | 0.775 |
| Kidney disease | 4 | 8 | 0.68 (0.18-2.53) | 9 | 6 | 1.71 (0.60-4.88) | 0.116 |
| Liver disease | 5 | 1 |  | 5 | 4 | 0.92 (0.24-3.52) | 0.443 |
| Gastrointestinal disease | 1 | 0 |  | 3 | 5 | 0.91 (0.25-3.29) |  |
| Lung disease | 2 | 3 | 0.33 (0.05-2.34) | 11 | 15 | 0.82 (0.35-1.94) | 0.690 |
| Thromboembolic disease^3^ | 1 | 4 |  | 8 | 18 | 0.47 (0.20-1.11) | 0.453 |
| Causes other than disease^4^ | 11 | 5 | 2.52 (0.79-8.07) | 35 | 20 | 2.01 (1.17-3.44) | 0.937 |
| Other or multiple conditions | 7 | 10 | 0.66 (0.24-1.82) | 16 | 12 | 1.43 (0.62-3.28) | 0.119 |
| Unknown cause of death | 3 | 2 | 1.88 (0.12-30.52) | 5 | 11 | 0.50 (0.17-1.47) | 0.391 |
| **TOTAL** | **181** | **168** | **0.83 (0.66-1.04)** | **413** | **515** | **0.80 (0.70-0.91)** | **0.641** |

Sub-hazard ratios (sHR) with 95% confidence intervals (CI) from Fine and Gray competing risk regression models (intention to treat-analysis). ^1^Adjusted for sex, age, BMI, smoking and year of inclusion. ^2^This includes aortic aneurysm, aortic thrombosis, diabetes gangrene, other large artery damage or multiple cardiovascular disease. ^3^This includes pulmonary embolism, vena cava thrombosis, other thromboembolic disease. ^4^This includes alcohol abuse, trauma, accident, suicide.

##### sFigures

**sFigure 1.** BMI during 20 years of follow-up in the T2D and non-T2D groups, stratified by intervention. Lines are estimated means from a mixed model with adjustment for sex and age

**sFigure 2. Survival in the surgery and control groups stratified by baseline type 2 diabetes status. Shown are subgroups with (dashed lines) and without (solid lines) baseline diabetes.** The graph shows the Kaplan–Meier estimate of survival (opaque lines) and the survival estimated from an unadjusted Gompertz regression model extrapolated up to 37.5 years (fainter lines). Per-protocol (as treated) analysis.
